# Robust positive control of tumour growth using angiogenic inhibition

**DOI:** 10.1049/syb2.12076

**Published:** 2023-10-03

**Authors:** Mohamadreza Homayounzade, Maryam Homayounzadeh, Mohammad Hassan Khooban

**Affiliations:** ^1^ Mechanical Engineering Department Fasa University Fasa Iran; ^2^ Aarhus University Aarhus Denmark; ^3^ Electrical Engineering Department Aarhus University Aarhus Denmark

**Keywords:** biocontrol, cancer, control theory, feedback, Lyapunov methods, nonlinear control systems, robust control

## Abstract

In practice, many physical systems, including physiological ones, can be considered whose input can take only positive quantities. However, most of the conventional control methods do not support the positivity of the main input data to the system. Furthermore, the parameters of these systems, similar to other non‐linear systems, are either not accurately identified or may change over time. Therefore, it is reasonable to design a controller that is robust against system uncertainties. A robust positive‐input control method is proposed for the automatic treatment of targeted anti‐angiogenic therapy implementing a recently published tumour growth model based on experiments conducted on mouse models. The backstepping (BS) approach is applied to design the positive input controller using sensory data of tumour volume as feedback. Unlike previous studies, the proposed controller only requires the measurement of tumour volume and does not require the measurement of inhibitor level. The exponential stability of the controlled system is proved mathematically using the Lyapunov theorem. As a result, the convergence rate of the tumour volume can be controlled, which is an important issue in cancer treatment. Moreover, the robustness of the system against parametric uncertainties is verified mathematically using the Lyapunov theorem. The real‐time simulation results‐based (OPAL‐RT) and comparisons with previous studies confirm the theoretical findings and effectiveness of the proposed method.

## Graphical abstract

A robust positive input control method is proposed for the automatic treatment of targeted anti‐angiogenic molecular therapy using a recently published tumour growth model based on mice experiments. The exponential stability of the controlled system and bounded input bounded output stability of the system in the presence of uncertainties are proved mathematically using the Lyapunov theorem. The actual input of the system is controlled without the need to extend the system model and generate dummy input to ensure the positivity of the input.

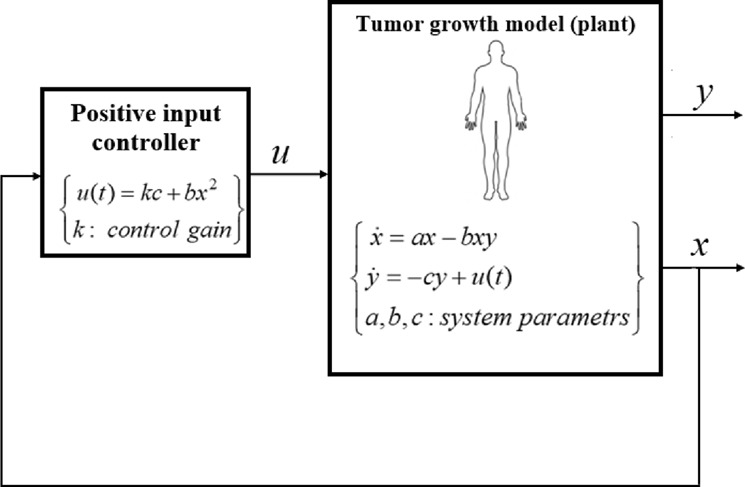

## 1 introduction

One of the challenges of control science is designing the controller for systems with positive input [1]. The input to positive systems is usually constrained to be non‐negative. However, conventional control methods are designed such that their input can take any value (i.e. positive or negative), so these methods cannot enforce the constraints imposed on the system input. The majority of physiological systems, such as blood glucose control systems [2, 3, 4, 5], tumour growth modelling systems [6, 7, 8, 9, 10, 11], chemical reaction systems [12] are positive input.

Positive input systems are very difficult to control because they are restricted to have an input with a positive magnitude all over the time. In addition, positive input systems similar to other non‐linear systems generally have intermittent variables, or their parameters vary over time, so it is necessary to use control methods that are robust against parametric uncertainties. Robust control of physiological systems has already been investigated by many authors in the literature, see for instance refs. [13, 14, 15, 16, 17, 18]. However, the positivity of the system input is not considered as an important issue during the controller design. In ref. [18], it has been tried to make the input of the system positive by adding saturation to the output of the controller. However, the positivity of the system has not been considered in the design phase.

In refs. [19, 20, 21, 22, 23, 24], the positivity of the input is considered in the controller design process. A virtual input is assumed for the system, and the system model is generalised by creating a bilinear differential equation that establishes a relationship between the actual and virtual system inputs such that for any value of the virtual input, the actual system input value always remains positive. As a result, the extended system is controlled by controlling the virtual input while ensuring the positivity of the actual input. However, if the system is extended, it will be non‐linear even if the primary system is linear. In ref. [22], feedback linearisation techniques are used to control the extended system by transforming the system into a set of integrators with infinite norms. In ref. [23], a robust‐norm‐based controller is designed for the tumour growth minimal model presented in refs. [10, 11]. In ref. [24], a control framework based on Linear Parameter Varying (LPV) method is designed. However, the methods presented in refs. [19, 20, 21, 22, 23, 24] suffer from the following shortcomings, which this article aims to overcome:

1. In refs. [19, 20, 21, 22, 23, 24], the actual system is extended by creating a bilinear differential equation that establishes a relationship between the real input (i.e. *u*) and virtual input (i.e. *v*) with the relation $\dot{u}=-uv$. The actual input *u* is then calculated by $u=u\left(0\right)\;\exp\left(-\int_{0}^{t}v\left(\tau \right)d\tau \right)$. Clearly for any magnitude of the virtual input, the system actual input is positive. Since there is no direct control over the actual input to the system, the value of *u*(*t*) may increase over time for certain values of *v*(*t*), for example, when the time history of the virtual input (i.e. $\int_{0}^{t}v\left(\tau \right)d\tau$) is incremented as a negative value. In this article, the actual input of the system is controlled without the need to extend the system model and generate virtual input. As a result, the actual input of the system can be directly controlled. Therefore, in contrary to refs. [19, 20, 21, 22, 23, 24], there is no possibility of the system input becoming unbounded.
2. In refs. [22, 23, 24], the controller only ensures *H*_*∞*_ or *H*_2_|*H*_*∞*_ stability of the system. In this paper, we analyse the stability of the equilibrium point and an exponentially stable robust controller for the tumour growth model is designed. In contrary to refs. [22, 23, 24] where the boundedness of the system state is considered, in this paper the exponential stability of the system is guaranteed and hence the convergence rate of system states can be controlled which is a very important issue in cancer treatment. Without the need to extend the system model, a robust controller with positive input is designed based on the BS approach. BS is a control approach that provides a recursive method to stabilise the origin of a system in a strict‐feedback form. Using the BS technique, we can control all the system states.
3. In refs. [18, 19, 20, 21, 22, 23], the robustness of the system is analysed only through simulation. In this article, the robustness of the system is analysed mathematically using the Lyapunov theorem. It is proved that the controlled system in the presence of perturbations is bounded input‐bounded output (BIBO) stable. Furthermore, the robustness of the proposed controller is investigated through simulations in Section 5 with 10%–100% change in tumour growth variables (tumour cell proliferation rate and drug efficacy). The simulation results confirm that the positive input is preserved, and the closed‐loop system remains robust against parametric uncertainties.
4. In refs. [22, 23], the control gain is designed using the iteration method to minimise the norm of the closed‐loop system. In ref. [24], based on the LPV methodology the parameter dependent feedback gain matrix is designed. Consequently, designing the control gain is difficult. In this paper, the control structure is much simpler compared to refs. [22, 23, 24], and therefore the control gain can be designed very simply.
5. In refs. [22, 23], the controllers require the measurement of the tumour volume and the inhibitor level and in refs. [19, 20, 21] in addition to the tumour volume and the inhibitor level, the controller requires the measurement of inhibitor injection rate. In ref. [24], a stabilising discrete extended Kalman filter is designed to estimate the inhibitor level. A stabilising controller is designed with the assumption that all system states are measurable, and then the inhibitor level estimate is used as the output of the estimation filter instead of the actual state in the control law. In fact, in ref. [24] the stability of the controller and estimating filter are analysed separately. As exactly mentioned in ref. [25], analysing the stability of the observer and controller separately may make the closed‐loop system unstable for some initial conditions. The proposed controller only needs to measure the tumour volume and does not need a filter to estimate the inhibitory level.

The rest of the paper is organised as follows: In Section 2, the system modelling in state space is presented and the error systems are presented. In Section 3, the BS controller is designed using the BS approach. In Section 4, the stability of the system is analysed using the Lyapunov theorem. In Section 5, the theoretical results are confirmed numerically using simulation results. Furthermore, the results are compared with those presented in refs. [22, 23, 24]. Finally, Section 6 concludes the results.

## 2 tumour growth model

In this section, first, the tumour growth model is presented in the state space, and then the error systems are presented.

### 2.1 System modelling

The differential equations governing the tumour growth model are calculated by the following [19, 20, 21, 22, 23, 24]:

(1a) $$\dot{x}=ax-bxy,$$

(1b) $$\dot{y}=-cy+u\left(t\right),$$

in which state *x* represents the time function of the tumour volume in *mm* ^3^, *y* represents the time function of the inhibitor level in *mg*/*kg*, *u* represents the time function of the inhibitor injection rate in *mg/kg/day*, parameter *a* denotes the proliferation rate of the tumour in 1/*day* which determines the rate of tumour cell division, the parameter *b* is the inhibition given in *kg*/*mg*/*day* which indicates the effectiveness of the utilised drug, and *c* denotes the clearance of the drug in 1/*day* which determines the rate of drug depletion.

The model is based on the fact that by using angiogenic inhibition the speed of tumour growth can be decreased. Equation (1a) describes the phenomenology of a tumour growth slowdown, as the tumour grows and resorts to its available support. The model describes the unstable dynamics of the limitless increase of non‐treated tumour concourse according to the *a* growth rate, while the tumour cell population—drug interaction causes the decrease of the tumour volume with rate *b*. The drug is depleted with the clearance rate *c*. This simplified model uses zero‐order pharmacokinetics, instead of the first‐order pharmacokinetics of the original model [26]; hence, the input of the simplified model is the serum level of the inhibitor.

### 2.2 Error systems

In this section, first, the design procedure of the controller using the BS technique is described, and then the proposed controller is presented in Theorem 1.

Let us define the variable *e* by

(2) $$e=y-y^{*},$$

where *y* ^∗^ represents the desired magnitude of *y* which will be designed properly in Section 3 using the BS approach. Considering the definition of *e* by Equation (2), we can rewrite Equation (1a) as follows:

(3) $$\begin{array}{l} \dot{x}=ax-bx\left(y-y^{*}+y^{*}\right),\\ =ax-bx\left(e+y^{*}\right). \end{array}$$

As a result, *y* ^*^ can act as a guiding variable for the controlling state *x*. In Section 3, the desired magnitude *y* ^*^ is designed properly using the BS approach such that the state *x* asymptotically converges to zero if *y* tends to *y* ^*^.

Differentiating the definition calculated by Equation (2), we obtain

(4) $$\dot{e}=\dot{y}-{\dot{y}}^{*}.$$

Substituting Equation (1b) in the result, we obtain

(5) $$\dot{e}=-cy+u\left(t\right)-{\dot{y}}^{*}.$$

Consequently, the control input should be designed in a way that the state *e* converges to zero, that is, *y* tends to *y* ^*^.

The backstepping approach provides a recursive method for stabilising the origin of a system in strict‐feedback form. Consider the system (1a), (1b), that is, the backstepping‐designed control input *u* has its most immediate stabilising impact on state *y*. Considering Equation (3), the states *y* then acts like a stabilising control individually on the state *x*. Hence *y* ^*^ (the desired magnitude of *y*) is designed to stabilise the state *x*. The backstepping approach determines how to stabilise the *x* subsystem using *y* ^*^, and then considering the equation governing *e* as Equation (5), the backstepping proceeds with determining how to design control law *u* to drive *e* converges to zero, that is, $\lim_{t\to \infty }y=y^{*}$.

The controller method using the BS technique is as follows:

1. The desired variable *y* ^*^ is appropriately designed such that the state *x* exponentially converges to zero if *y* tends to *y* ^*^.
2. The input control law *u* is appropriately designed so that the tracking error *e* exponentially converges to zero, that is, *y* tends to *y* ^*^.

## 3 CONTROLLER DESIGN

The BS technique is used to design the desired magnitude *y* ^*^ as follows:

(6) $$y^{*}=k,$$

where the constant *k* is the positive control gain.

Substituting *y* ^*^ by Equation (6) in Equation (3), we obtain

(7) $$\dot{x}=\left(a-bk\right)\;x-bxe.$$

Consequently, if the control gain is designed such that

(8) $$k>\frac{a}{b},$$

we have

(9) $$\dot{x}=-dx-bxe,$$

where *d* = *bk* − *a* is a positive constant.

Let us design the control input by

(10) $$u\left(t\right)=cy^{*}+bx^{2},$$

where *y* ^*^ is defined previously by Equation (6).

**FIGURE 1:**
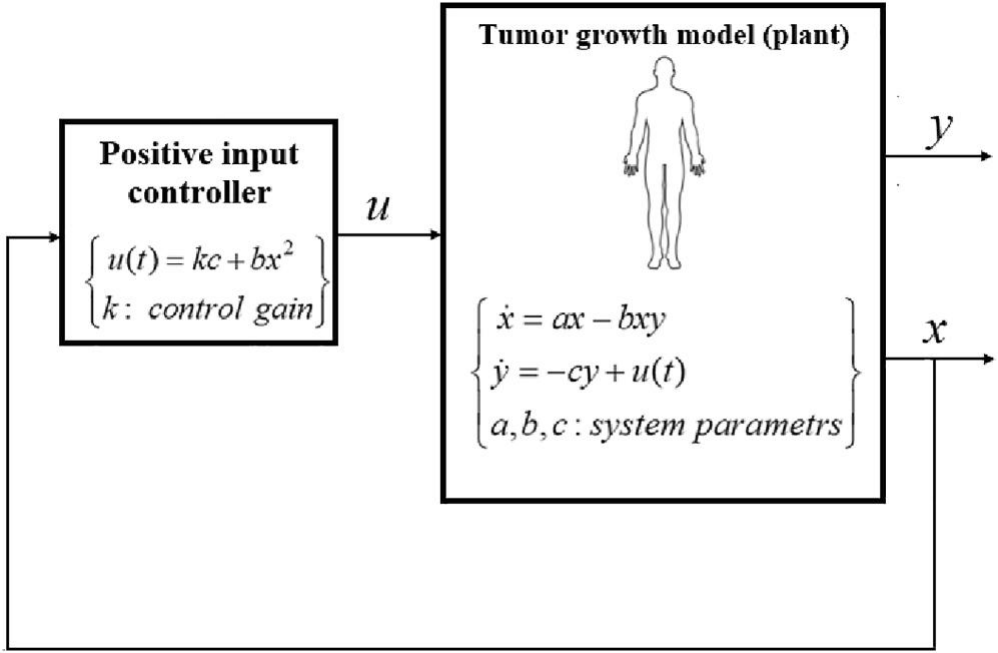
The block diagram schematic of the controlled system.

The schematic of the closed‐loop controlled system is shown in Figure 1.

Remark 1. Considering Equation (6), it is clear that *y* ^*^ is a positive signal. Moreover, the term *b* *x* ^2^ is always non‐negative, hence it can be stated that the control input *u* *(* *t* *)* calculated by Equation (10) is always positive.

Remark 2. It is worth mentioning that unlike [22, 23] in which the controller requires the measurement of states *x*, *y* the proposed controller requires only the measurement of state *x* as feedback.

Substituting the control input by Equation (10) in the error system by Equation (5) and considering that ${\dot{y}}^{*}=0$, we obtain

(11) $$\begin{array}{l} \dot{e}=-c\left(y-y^{*}\right)+bx^{2},\\ =-ce+bx^{2}. \end{array}$$

Theorem 1. *The control input calculated by Equation* (10) *exponentially stabilises the system such that the state x and e exponentially converges to zero*, that is, $\left|x\left(t\right)\right|\leq \beta e^{-\frac{1}{2}\;\gamma t},$ $\left|e\left(t\right)\right|\leq \beta e^{-\frac{1}{2}\;\gamma t},$ *where the positive constant γ adjusts the rate of convergence of system errors*. *It will be shown that by increasing the control gain k*, *the rate of convergence of tumour volumes and inhibitor level decreases*.

## 4 stability analysis

In this section, first, we analyse the equilibrium point stability. Then, the BIBO stability of the controlled system in the presence of uncertainties in the system parameters is analysed using the Lyapunov theorem.

### 4.1 Stability of the equilibrium point

Choose the following Lyapunov function candidate:

(12) $$V=\frac{1}{2}x^{2}+\frac{1}{2}e^{2}.$$

Differentiating Equation (12) with respect to time to obtain

(13) $$\dot{V}=x\;\dot{x}+e\;\dot{e}.$$

Substituting Equations (9) and (11) in Equation (13) to obtain

(14) $$\dot{V}=x\left(-dx-bxe\right)+e\left(-ce+bx^{2}\right).$$

Simplifying Equation (14) we obtain

(15)

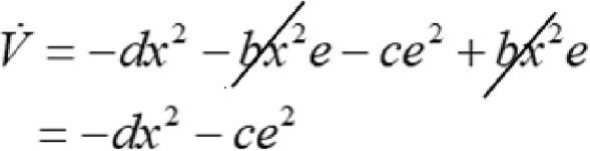




Consequently, we have

(16) $$\dot{V}\leq -\gamma V,$$

where *γ* = 2 min{*d*,*c*} is a positive constant. Equation (16) implies that $\dot{V}\leq 0$.

Then integrate both sides of Equation (16) to find

(17) $$\int_{V\left(0\right)}^{V\left(t\right)}\frac{dV}{V}\leq -\gamma \int_{0}^{t}dt\;\Rightarrow \mathrm{Ln}\left(\frac{V\left(t\right)}{V\left(0\right)}\right)\leq -\gamma t,$$

as well

(18) $$V\left(t\right)\leq V\left(0\right)\;e^{-\gamma t}.$$

Let us define the augmented state *X* as

(19) $$X=\left[\begin{array}{l} x\\ e \end{array}\right].$$

Considering the definition calculated by Equation (19), the Lyapunov function can be considered as follows:

(20) $$V\left(t\right)=\frac{1}{2}X^{T}X.$$

Let us define the Euclidean norm for an arbitrary vector *X* as

(21) $${\left\|X\right\|}^{2}=X^{T}X.$$

Considering Equations (20) and (21), the Equation (18) can be considered as follows:

(22) $$\left\|X\left(t\right)\right\|\leq \left\|X\left(0\right)\right\|\;e^{-\frac{1}{2}\gamma t}.$$

Considering definition calculated by Equation (19) and Equation (22) results

(23) $$\begin{array}{l} \left|x\left(t\right)\right|\leq \beta \;e^{-\frac{1}{2}\gamma t},\\ \left|e\left(t\right)\right|\leq \beta \;e^{-\frac{1}{2}\gamma t}, \end{array}$$

where $\beta \geq \sqrt{x^{2}\left(0\right)+e^{2}\left(0\right)}$ is a positive constant. Equation (23) implies that states *x*(*t*) and *e*(*t*) exponentially converge to zero, that is,

(24) $$\lim_{t\to \infty }\;x\left(t\right)=0,\lim_{t\to \infty }\;e\left(t\right)=0.$$

Remark 3. Considering Equation (23) it can be observed that the parameter *γ* specifies the rate of convergence of *x(t)*, *e(t)*. Consequently, by adjusting the control gain *k*, we can adjust the parameter *d* and as a consequence, we can control the rate of convergence of state *x*.

### 4.2 Robustness of the closed‐loop system

In this subsection, the robustness of the closed‐loop system is analysed in the presence of parametric uncertainties. Let the nominal value of system parameters be defined by “¯” notation.

Theorem 2. *For the tumour growth system modelled by Equations* (1a), (1b), *the controller given by Equation* (25) *ensures BIBO stability of the system in the presence of parametric uncertainties.*

(25) $$u\left(t\right)=\bar{c}y^{*}+\bar{b}x^{2},$$

Following a method similar to that presented in Section 3, the system error dynamics in the presence of parametric uncertainties are given by

(26a) $$\dot{x}=\left(a-bk\right)x-bxe,$$

(26b) $$\dot{e}=-\bar{c}e+\bar{b}x^{2}+u_{d}\left(t\right),$$

where *u*_*d*_(*t*) lumped the deviation of system parameters from their nominal magnitudes, that is, $u_{d}\left(t\right)=-\left(c-\bar{c}\right)e+\left(b-\bar{b}\right)x^{2}$.

For all trajectories within the region *Ω* = {*X*(*t*)| ‖*X*(*t*)‖ < *δ*}, it can be stated that the perturbation *u*_*d*_(*t*) is bounded (i.e. |*u*_*d*_(*t*)|< *δ*_*d*_), where *δ*, *δ*_*d*_ are positive constants.

Consider the Lyapunov function as follows:

(27) $$V=\frac{1}{2}X^{T}X,$$

where the augmented state *X* was previously defined by Equation (19). Considering the system errors in the presence of uncertainties calculated by Equations (26a) and (26b) and take *δ* > 0 and *δ*_*d*_ > 0 such that *Ω* = {*X*(*t*)| ‖*X*(*t*)‖ ≤ *δ*} and *Ω*_*d*_ = {*u*_*d*_(*t*)| ‖*u*_*d*_(*t*)‖≤*δ*_*d*_}, where *Ω*⊂*R* ^2^ is the domain contains *x* = 0 and *Ω*_*d*_ ⊂ *R* is the domain contains*u*_*d*_ = 0.

Considering the Equations (26a) and (26b) and following the same method as that presented in Section 4.1, the time derivative of the Lyapunov function is calculated by

(28) $$\dot{V}=-d\;x^{2}-\bar{c}e^{2}+eu_{d}\left(t\right),$$

where *d* = *bk*−*a* is a positive constant.

Consequently, for all (*t*, *x*, *u*_*d*_) ∈ [0,*∞*) × *Ω* × *Ω*_*d*_ we obtain

(29) $$\begin{array}{l} \dot{V}\leq -\bar{\gamma }\;{\left\|X\left(t\right)\right\|}^{2}+\left\|X\left(t\right)\right\|\;\left\|u_{d}\left(t\right)\right\|\\ \leq -\bar{\gamma }\;{\left\|X\left(t\right)\right\|}^{2}+\delta_{d}\;\left\|X\left(t\right)\right\|, \end{array}$$

where $\bar{\gamma }=\min\left\{d,\bar{c}\right\}$. Consider $W=\sqrt{V\left(x\right)}$, when *V*(*x*) ≠ 0, use W˙=V˙2V and Equation (29) and consider Equations (21) and (27) to obtain

(30) $$\begin{array}{l} \dot{W}\leq \frac{-\bar{\gamma }\;{\left\|X\left(t\right)\right\|}^{2}+\left\|X\left(t\right)\right\|\delta_{d}}{\sqrt{2}\;\left\|X\left(t\right)\right\|}\\ \leq -\frac{1}{\sqrt{2}}\bar{\gamma }\;\left\|X\left(t\right)\right\|+\frac{1}{\sqrt{2}}\delta_{d}\\ \leq -\gamma \;W+\frac{1}{\sqrt{2}}\delta_{d}. \end{array}$$

It is shown in the Appendix that when *V*(*X*) = 0 it can be verified that

(31) $$D^{+}W\leq \frac{1}{\sqrt{2}}\delta_{d}.$$

Hence for all magnitudes of *V*(*X*) we have,

(32) $$D^{+}W\leq -\bar{\gamma }W+\frac{1}{\sqrt{2}}\left\|u_{d}\left(t\right)\right\|.$$

Let us mention the Lemma known by comparison Lemma.

*Consider the scalar differential equation*

(33) $$\dot{\xi }=h\left(t,\xi \right),\;u\left(t_{0}\right)=\xi_{0},$$

*where h* (*t*,*ξ*) *is continuous in* *t* *and locally Lipschitz in* *ξ*, *for all* *t* ≥ 0 *and all ξ* ∈ *J* ⊂ *R*. *Let* *t* ∈ [*t*_0_,*T*) (*T* *could be infinity) be the maximal interval of existence of the solution* *ξ*(*t*), *and suppose* *ξ*(*t*) ∈ *J* *for all* *t* ∈ [*t*_0_,*T*). *Let* *η*(*t*) *be a continuous function whose upper right‐hand derivative* *D* ^+^ *η*(*t*) *satisfies the differential inequality*

(34) $$D^{+}\eta \left(t\right)\leq h\left(t,\eta \left(t\right)\right),\eta \left(t_{0}\right)\leq \xi_{0},$$

*with* *η*(*t*) ∈ *J* *for all* *t* ∈ [*t*_0_,*T*). *Then*, *η*(*t*) ≤ *ξ*(*t*) *for all* *t* ∈ [*t*_0_,*T*).

Using the comparison Lemma, we can then find

(35) $$\begin{array}{l} W\left(t\right)\leq e^{-\bar{\gamma }t}\;W\left(0\right)+\frac{1}{2}\int_{0}^{t}e^{-\bar{\gamma }\left(t-\tau \right)}\left\|u_{d}\left(\tau \right)\right\|\;d\tau .\\ \leq e^{-\bar{\gamma }t}\;W\left(0\right)+\frac{1}{2\gamma }\delta_{d}\left(1-e^{-\bar{\gamma }t}\right). \end{array}$$

Using Equations (21) and (27), we obtain

(36) $$\left\|X\left(t\right)\right\|\leq \left\|X\left(0\right)\right\|e^{-\bar{\gamma }t}+\frac{1}{\sqrt{2}\gamma }\delta_{d}\left(1-e^{-\bar{\gamma }t}\right).$$

It can be easily verified that Equation (37) guarantees ‖*X*(*t*)‖≤*δ*; as a result, the state *x*(*t*) remains within the domain of validity of the assumptions. Furthermore, the origin is globally exponentially stable and all the assumptions hold globally with *Ω*∈*R* ^2^. Consequently, the robustness of the system is assured using a high gain controller. The proposed BS controller is summarised in Table 1.

(37) $$\left\|X\left(0\right)\right\|\leq \delta ,\underset{0\leq \sigma \leq t}{\sup}\;\left\|u_{d}\left(\sigma \right)\right\|\leq \delta_{d},$$

**TABLE 1:** BS controller design.

The tumour growth model:	$\left\{\begin{array}{l} \dot{x}=ax-bxy,\\ \dot{y}=-cy+u\left(t\right), \end{array}\right.$ *x*: time function of the tumour volume, *y*: time function of the inhibitor level.
Input controller:	*u*(*t*) = *kc* + *bx* ^2^, *k*: Control gain.
Stability:	Exponentially stable, BIBO stable.
Restriction:	The control gain should be designed $k>\frac{a}{b}.$

## 5 simulation results

In this section, in order to verify the pre‐eminence of the proposed control method for the tumour growth modelled by Equations (1a) and (1b) in MATLAB/Simulink software. The relevant parameters of the concerned system are given in Table 2.

**TABLE 2:** System parameters.

Growth rate of the tumour:	*a* = 0.27 1/*day*
Inhibition:	*b* = 0.0074 *kg*/*mg*/*day*
Clearance of the drug:	*c* = ln (2)/3.9 1/*day*

Besides, the HIL simulator is employed to explore the pertinence of the proposed scheme to the context of the tumour growth model.

Hardware‐in‐the‐loop (HIL) refers to a technique that is used in the development and testing of complex real‐time embedded systems. The real‐time analysis, which the HIL method performs, allows for considering errors and delays that are commonly dismissed in offline MATLAB simulations. In HIL simulation, we use a real‐time computer as a virtual representation of our plant model and a real version of your controller. To apply the HIL method on the proposed system, a real‐time simulator was used that encompassed both a controller and plant. The Simulink model of the overall system, encompassing the proposed controller, was further modified and compiled with the help of MATLAB and OPAL RT‐Lab library to adjust it to HIL technology. In compilation process, the overall system was divided into three subsystems, namely primary, secondary, and console, for RT‐Lab simulation. The primary system consisted of the medical case study, excluding the controllers and the scope, which were kept in the secondary subsystem while the visual output equipment such as scopes were preserved in the console subsystem. Subsequently, the whole model, with all its subsystems, was loaded to the OPAL‐RT server to convert it to the corresponding “C” code of the model under test. Keeping the solver time step in the fixed‐step mode of a real‐time system, the simulation was performed [27, 28].

We simulate the proposed control method for the tumour growth modelled by Equations (1a) and (1b). The most similar researches in the literature are refs. [22, 23, 24]. The proposed method has some advantages compared to other similar methods in the literature as discussed in Table 3. In Section 5.1, the results of the proposed method are compared with the results presented in ref. [24]. Furthermore, in Section 5.2, we compare our results with those presented in refs. [22, 23].

**TABLE 3:** Comparison between proposed control method and [22, 23, 24].

Comparison	Control law	Discussion
This paper	✓ The actual input of the system is controlled. ✓ The controller requires only measurement of time function of the tumour volume. ✓ Exponential stability is guaranteed. ✓ The BIBO stability of the system against uncertainties is proved mathematically.	✓ The actual value of the system input can be directly controlled and there is no possibility for the system input to become unbounded. ✓ The controller eliminates the need to measurement of the time function of the inhibitor level. ✓ The controller improves the control performance such that the rate of convergence of tumour volume can be controlled. ✓ The control gain can be easily designed and controller structure is very simple. ✓ The robustness of the system against uncertainties has been mathematically and numerically ensured.
Ref. [22, 23]	✓ The model is extended and the virtual input is controlled to ensure that the actual input is positive. ✓ The controller only guarantees the *H*_*∞*_ stability. ✓ The robustness of the system has only been analysed through simulation. ✓ Control gain should be designed using iteration method. ✓ The controller needs to measure the time function of the inhibitor level in addition to the time function of the tumour volume.	✓ There is no direct control over the actual input to the system. ✓ The control gain design is difficult and also the controller structure is complex. ✓ The controller only guarantees the boundedness of *L*_*∞*_‐norm of the tumour volume. ✓ The robustness of the system against uncertainties is shown only through simulation.
Ref. [24]	✓ The model is extended and the virtual input is controlled to ensure that the actual input is positive. ✓ The controller only ensures *H*_2_|*H*_*∞*_. ✓ The robustness of the system has only been analysed through simulation. ✓ The feedback gain matrix is designed based on the LPV method.	✓ There is no strict control over the actual input to the system. ✓ The control gain design is difficult and the controller structure is complex. ✓ The controller only guarantees the boundedness of *L*_2_‐norm of tumour volume. ✓ The robustness of the system against uncertainties is shown only through simulation.

### 5.1 Stability and robustness analysis against uncertainties

In this section, initially we simulate the system modelled by Equations (1a) and (1b) with system parameters presented in Table 2 similar to refs. [22, 23, 24].

In ref. [22], the positive input dynamics methodology is developed such that the positive system is transformed into a linear system that can be used for *H*_*∞*_ norm‐based robust controller design for the tumour growth minimal model. The control law is calculated by

(38) $$u=K_{0}\tilde{\phi }+0.125\left(K_{r}\left(s\right)r\left(s\right)-K_{y}\left(s\right)y\left(s\right)\right),$$

where *r*(*s*) is the Laplace transform of the reference signal, *K*_0_ is defined in Table 4 and

(39) $$\tilde{\phi }=\left[\begin{array}{c} x\\ ax-bxy\\ \left({\left(a-by\right)}^{2}-b\left(u-cy\right)\right)x \end{array}\right].$$

**TABLE 4:** Controller parameters.

Tumour volume initial condition:	10^4^ *mm* ^3^
Inhibitor level initial condition:	0mgkg
Control gain (proposed method), that is, *K*:	60
Fixed parameter vector (method proposed in ref. [24]), that is, *p*_*b*_:	[10^−3^,*a*/*b*+10^−3^,0,10^−5^]
The eigenvalues of the open system (method proposed in ref. [24]), that is, *λ*(*A* (*p*_*b*_)):	[0.2699,−0.1777,0]^*T*^
Final parameter dependent feedback gain (method proposed in ref. [24]), that is, *K* (*p*_*b*_):	[5.554,8.5339,3.3666]^*T*^
Feedback gain (method proposed in ref. [22]), that is, *K*_0_:	[0.125 0.75 1.5]

In ref. [24], a general state‐feedback controller in LPV sense has been realised as follows:

(40) $$u\left(t\right)=r\left(t\right)-K\left(p\left(t\right)\right)\bar{X}\left(t\right),$$

where *r*(*t*) represents the reference signal and $\bar{X}={\left[x,y,v\right]}^{T}$ and *v* is the virtual input calculated from $\dot{u}=-vu$ and *K*(*p*(*t*)) is parameter‐dependent feedback gain matrix. The LPV representation of system model is then calculated by

(41) $$\begin{array}{l} \dot{\bar{X}}\left(t\right)=A\left(p\left(t\right)\right)\bar{X}\left(t\right)+B\left(p\left(t\right)\right)u\left(t\right),\\ y\left(t\right)=C\left(p\left(t\right)\right)\bar{X}\left(t\right)+D\left(p\left(t\right)\right)u\left(t\right). \end{array}$$

The feedback gain is then calculated by

(42) $$K\left(p\left(t\right)\right)=-B{\left(p\left(t\right)\right)}^{+}\left(A\left(p_{b}\right)-A\left(p\left(t\right)\right)-B\left(p_{b}\right)K\left(p_{b}\right)\right)$$

where *K*(*p*_*b*_) represents the final parameter‐dependent feedback gain presented in Table 4.

The initial conditions of system states in the simulation and the control gains are presented in Table 4.

**FIGURE 2:**
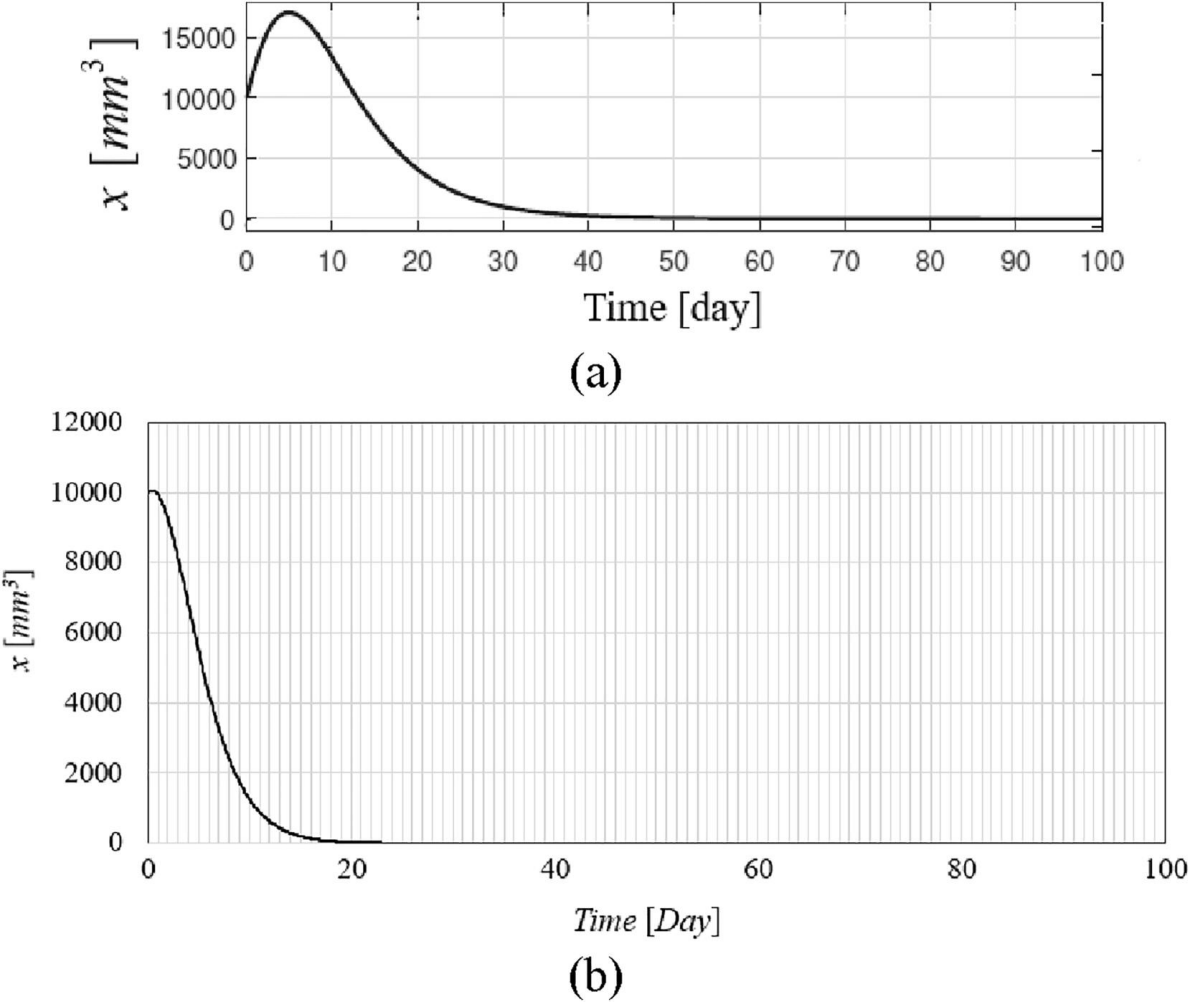
The time history of tumour volume with initial condition presented in Table 4, (a) The controller proposed in ref. [24], (b) The proposed control method.

**FIGURE 3:**
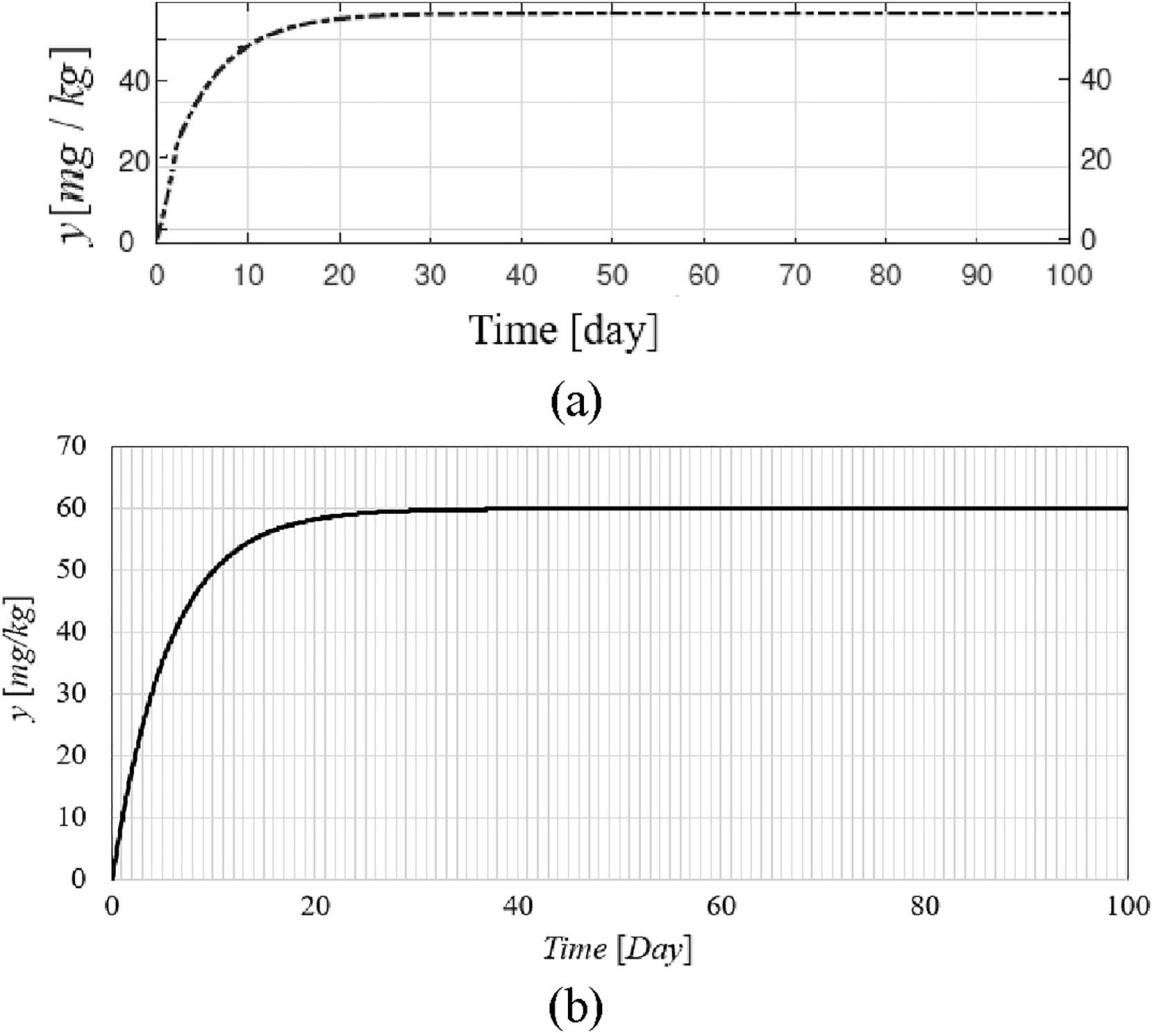
The time history of inhibitor level with initial condition presented in Table 4, (a) The controller proposed in ref. [24], (b) The proposed control method.

**FIGURE 4:**
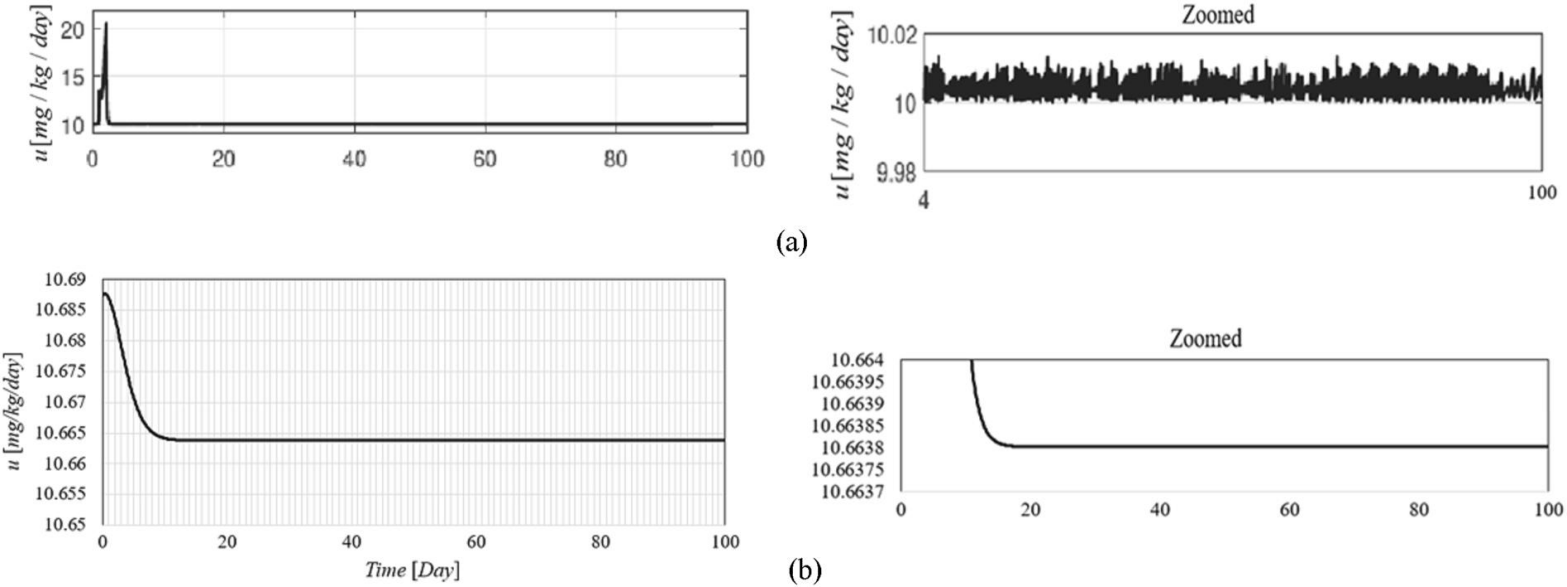
The time history of injection rate, (a) The controller proposed in ref. [24], (b) The proposed control method.

The time history of resulting tumour volume, inhibitor level, and the injection rate for the proposed control method and those presented in ref. [24] are shown in Figures 2, 3, 4. From Figure 2, it can be seen that with the proposed method, the tumour volume increases by 7% at the beginning of treatment, while it increases by 70% in ref. [24]. This is justified because, considering Figure 3, the inhibition level is initially very low, and when the inhibition level increases, the tumour growth slows down.

From Figure 4, it can be seen that at first there is a peak in the rate of inhibitor injection to reduce the growth of tumour volume, and then, as the tumour volume decreases, the injection rate decreases. The injection rate is initially high and reaches a constant value after the first large injection (Figure 4). It can be seen that the injection rate of the method proposed in ref. [24] fluctuates greatly because of the state prediction error, which may reduce the practical efficiency of the controller. From Figure 2, it can be seen that the initial peak in the tumour growth volume is lower compared to the initial peak in ref. [24]. This is justified because the proposed controller guarantees the stability of the equilibrium point, hence the peak value of the tumour growth can be reduced by adjusting the control gains.

**FIGURE 5:**
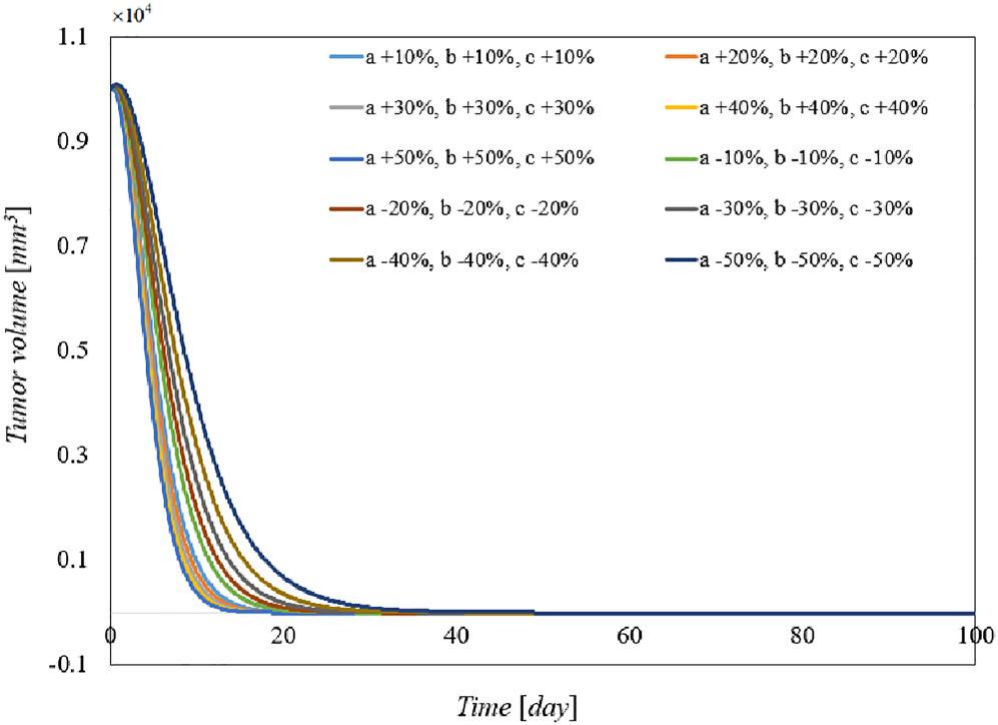
The time history of tumour volume with perturbation of the model parameters *a*, *b*, *c*.

**FIGURE 6:**
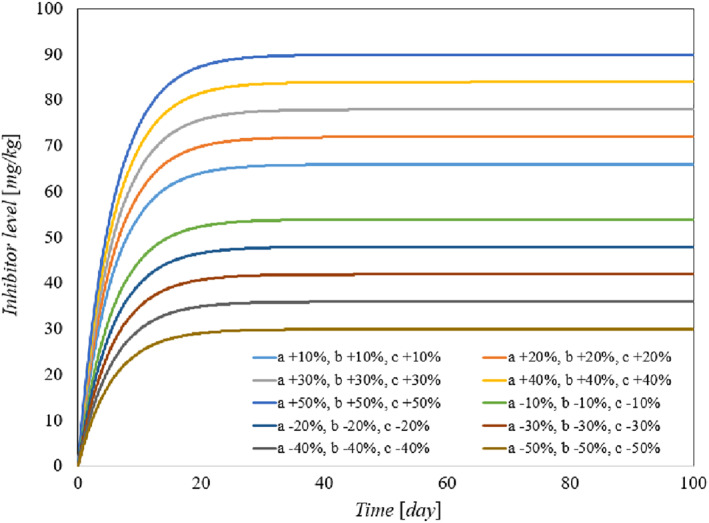
The time history of inhibitor level with perturbation of the model parameters *a*, *b*, *c*.

**FIGURE 7:**
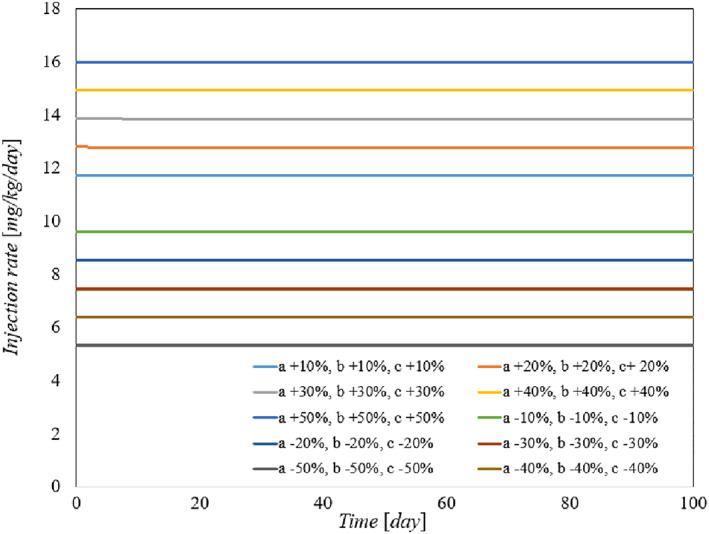
The time history of injection rate with perturbation of the model parameters *a*, *b*, *c*.

To demonstrate the robustness of the system against parameter uncertainties, we simulate the proposed controller against 10%–100% uncertainties in the system parameters. The time history of the resulting tumour volume, inhibitor level, and injection rate are shown individually in Figures 5, 6, 7. It can be seen that the controller is robust against uncertainties in system parameters. In addition, it is observed that a higher control injection rate is needed to control the system against more uncertainties in the system parameters.

**FIGURE 8:**
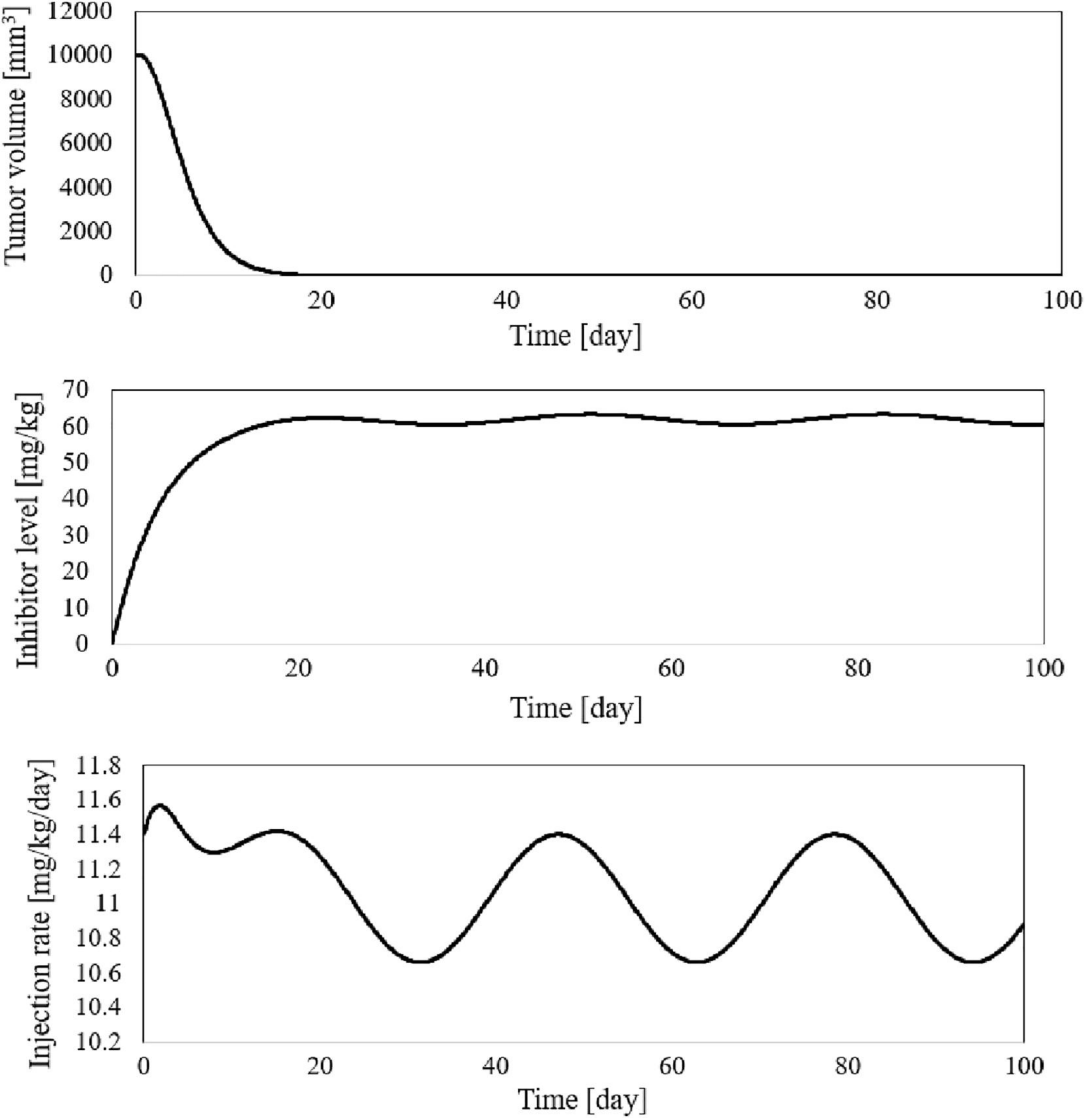
Time history of tumour volume, inhibitor level and injection rate in the presence of noise in tumour volume measurement.

As mentioned previously, the proposed controller requires only the measurement of tumour volume. To analyse the robustness of the controller against noise in tumour volume measurement, the controller is simulated considering the non‐constant noise in feedback measurement as $\bar{x}\left(t\right)=x\left(t\right)+30\;\sin\left(0.1\;t\right)$ where $\bar{x}\left(t\right)$ represents the measured tumour volume contaminated with noise and the amplitude of noise is considered to be 30 *mm* ^*3*^ which fluctuates with frequency of 0.1radsec. The control law is then calculated by $u\left(t\right)=ck+b{\bar{x}}^{2}$. The simulation results of the proposed controller against noise in measurements are shown in Figure 8.

**FIGURE 9:**
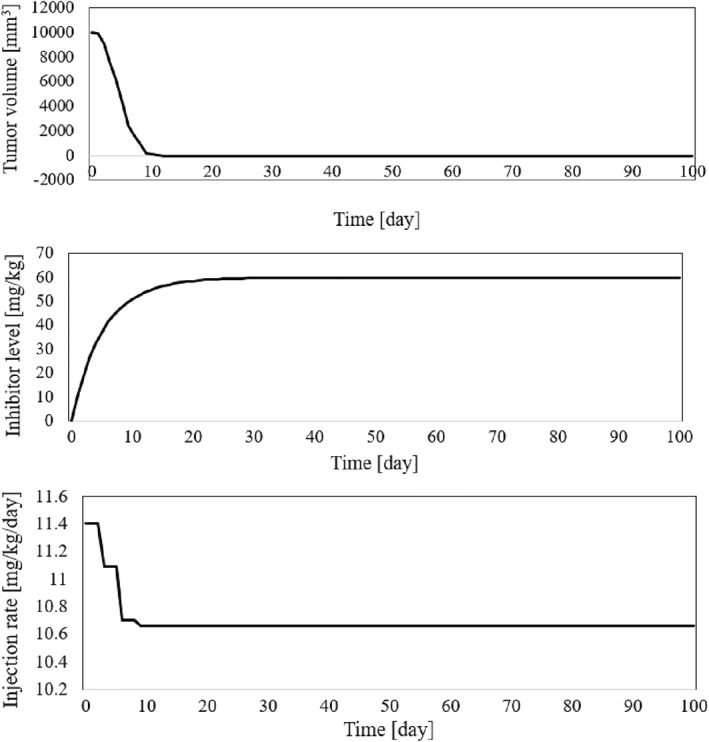
Time history of tumour volume, inhibitor level and injection rate in the presence of discrete measurement of tumour volume.

In practice the controller uses the discrete measurement of tumour volume, that is, $u\left(t\right)=ck+b{x_{t}}^{2},$ where *x*_*t*_ = (*x*(*t*), *x*(*t*−*l*_1_), *x*(*t*−*l*_2_), …, *x* (*t*−*l*_*m*_)) represents the discrete measurements of tumour volume and *l*_1_…*l*_*m*_ are state delays. Therefore, in the simulation section, we assumed that the tumour measurement is done every 3 days, and we simulated the controlled system using these discrete tumour volume measurements, and the results are presented in Figure 9.

### 5.2 Comparison results with [22, 23]

**FIGURE 10:**
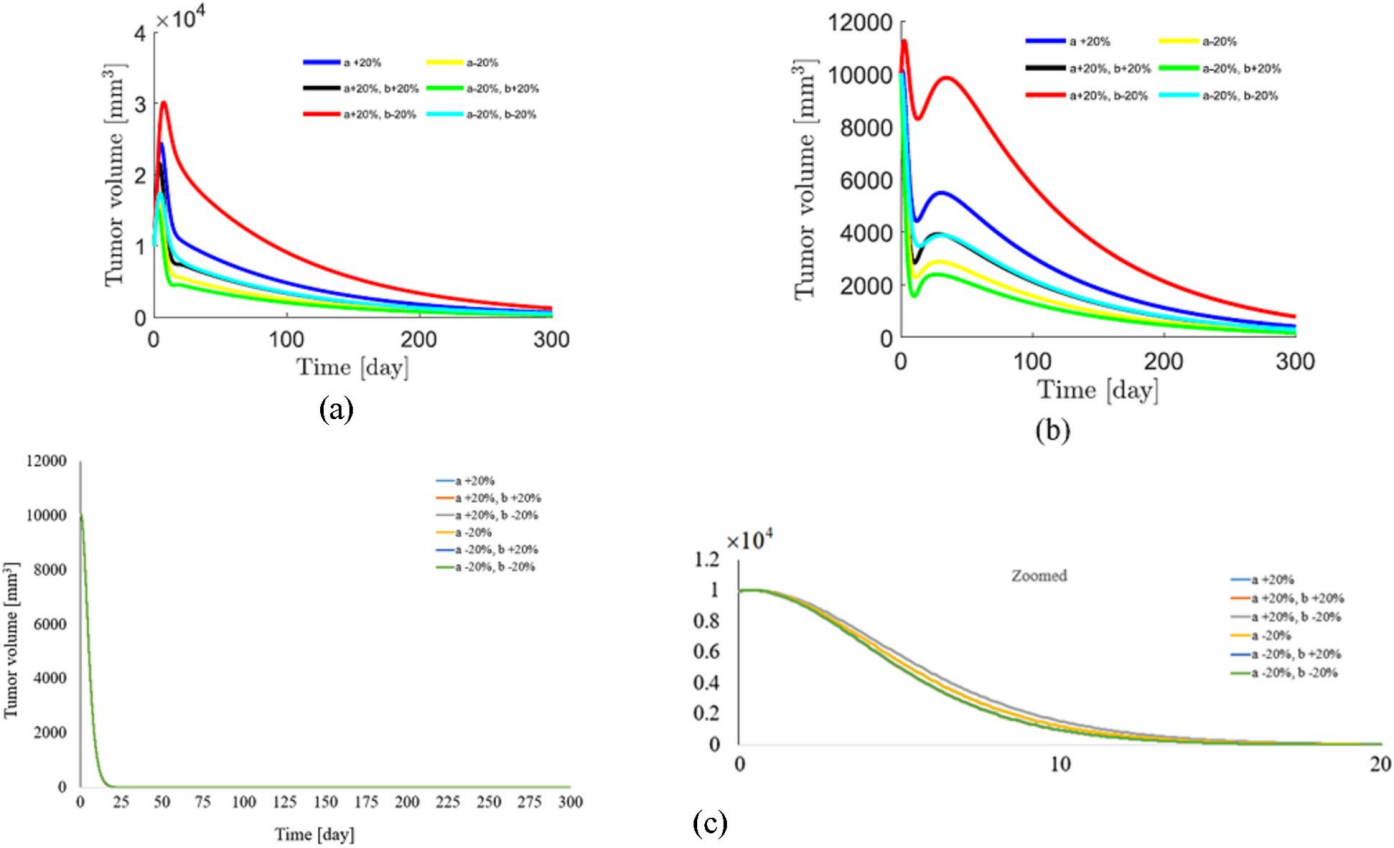
The time history of tumour level with perturbation of the model parameters *a*, *b*. (a) The controller proposed in ref. [22], (b) Controller proposed in ref. [23], (c) The proposed control method.

**FIGURE 11:**
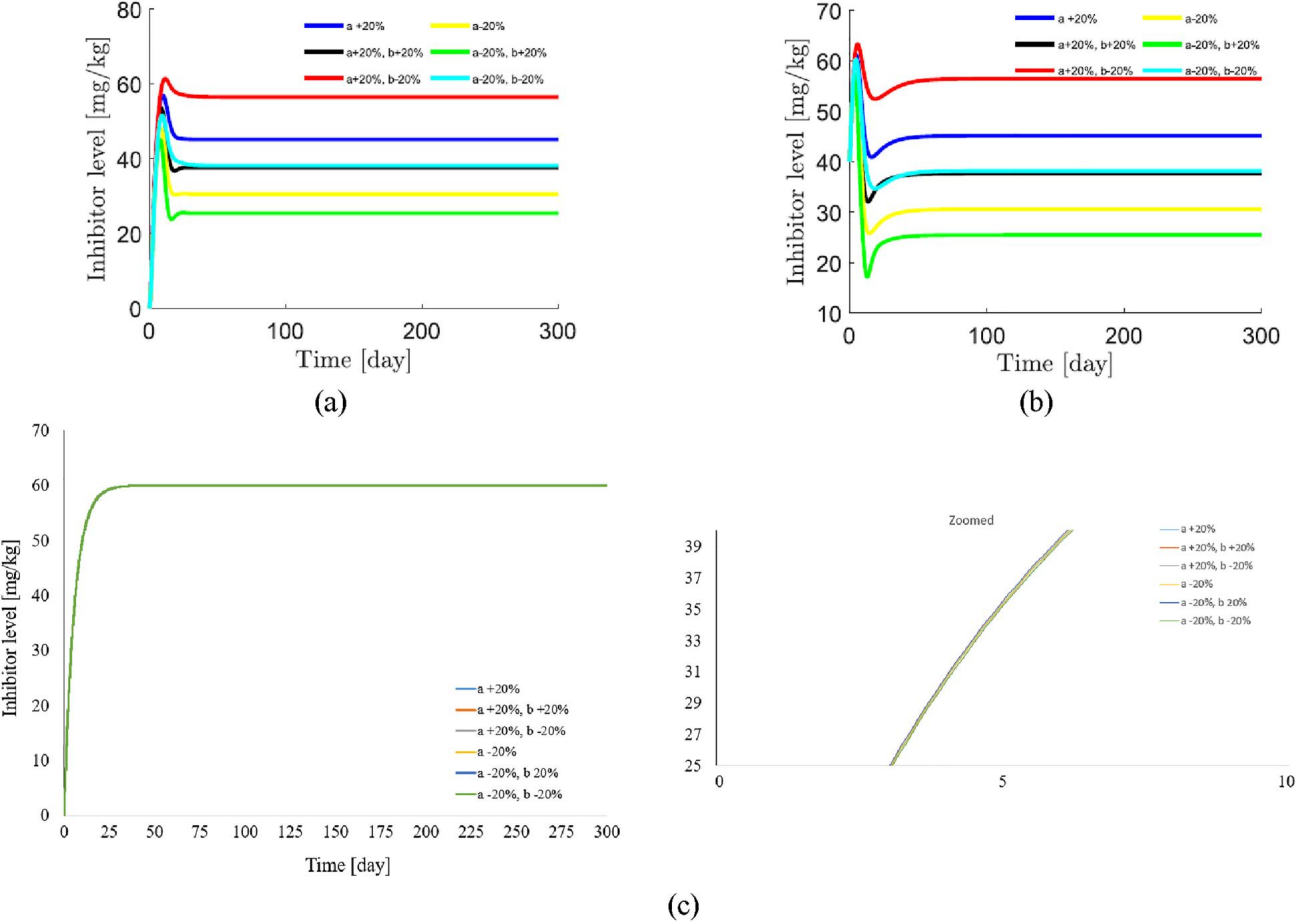
The time history of inhibitor level with perturbation of the model parameters *a*, *b*. (a) The controller proposed in ref. [22], (b) Controller proposed in ref. [23], (c) The proposed control method.

**FIGURE 12:**
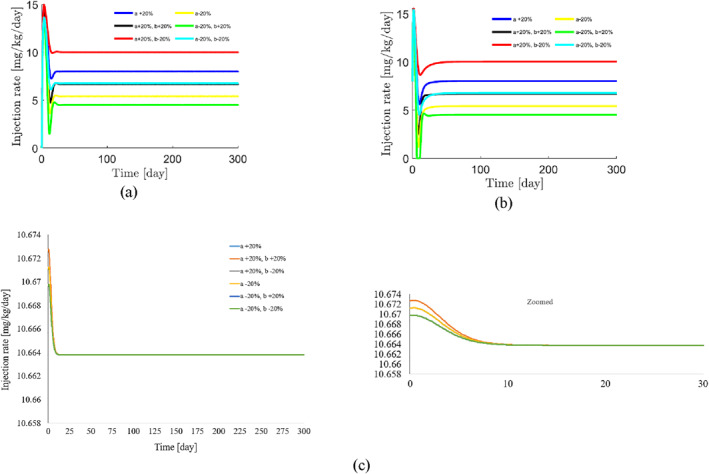
The time history of injection rate with perturbation of the model parameters *a*, *b*. (a) The controller proposed in ref. [22], (b) Controller proposed in ref. [23], (c) The proposed control method.

To show the effectiveness of the proposed control method, the results are compared with those presented in refs. [22, 23]. The time history of the resulting tumour volume, inhibitor level, and injection rate are shown in Figures 10, 11, 12.

From Figure 10, it can be seen that the error systems converge faster and more accurately using the proposed controller than [22, 23]. This is justified, because the proposed controller guarantees exponential stability, so the rate of convergence of the tumour volume and the inhibitor level can be controlled. In addition, the initial peak in the tumour growth volume is lower compared to the initial peak in refs. [22, 23]. This is justified because the proposed controller guarantees the stability of the equilibrium point, hence the peak value of the tumour growth can be reduced by adjusting the control gains.

The injection rate is initially high and reaches a constant value after the first large injection (Figure 12).

Considering Equation (10), it can be easily verified that the controller is completely robust against system parameter *a*. In other words, the change in parameter *a* has no effect on the change in control law *u*(*t*). However, as the parameter $\bar{b}$ changes the term $\bar{b}x^{2}$ also changes. In this paper, the controller ensures that $\lim_{t\to \infty }\;x=0$, and consequently $\lim_{t\to \infty }\;u\left(t\right)=\lim_{t\to \infty }k\bar{c}+\lim_{t\to \infty }\bar{b}x^{2}=k\bar{c}$. As a result, the steady‐state value of the control injection rate is robust against changes in the system parameters *a*,*b*, and only changes in the parameter *c* cause the value of the control law to change.

Figure 10 depicts the time history of the tumour volume in the presence of uncertainties in system parameters *a*,*b*. In the zoomed‐version it can be seen that uncertainties in parameter *a* has no effect on the time history of the state *x*. However the changes in parameter *b* has effect on the time history of state *x* in transient time.

In this paper, we prove that $\lim_{t\to \infty }\;y\left(t\right)=y^{*}=k$. Consequently, the steady magnitude of inhibitor level depends only on the control gain and not the system parameters. However, the parameter *b* affects the transient magnitude of inhibitor level and the parameter *a* has no effect on the time function of inhibitor level. Figure 11 confirms the above claims.

## 6 CONCLUSION

This study presented the development of positive input dynamics method for the tumour growth control. Unlike previous studies, the proposed method does not need to extend the system model to ensure the positivity of the system input. As a result, in this article, we control the actual input and not the virtual input, and therefore, unlike previous methods, using the proposed method, it is not possible for the actual input to be unbounded. The proposed controller only needs to measure the tumour volume, and the global exponential stability of the system was proved using the Lyapunov theorem. Due to the exponential stability, the convergence rate of the tumour volume can be controlled by adjusting the control gain. The robustness of the system against uncertainties was proved using the Lyapunov theorem and it was shown mathematically and numerically that the system is BIBO stable. The simulation results show the effectiveness and superiority of the proposed controller compared to previous related studies. As a future work, we will extend the proposed method to a more complex tumour models.

## AUTHOR CONTRIBUTIONS

**Mohamadreza Homayounzade**: Conceptualization; formal analysis; investigation; methodology; project administration; resources; software; validation; writing—original draft; writing—review and editing. **Maryam Homayounzadeh**: Conceptualization; investigation; methodology; project administration; resources; supervision; writing—original draft; writing—review and editing. **Mohammad Hassan Khooban**: Data curation; investigation; methodology; project administration; software; supervision.

## CONFLICT OF INTEREST STATEMENT

All authors declare that they have no conflicts of interest.

## Data Availability

The data that support the findings of this study are available from the corresponding author upon reasonable request.

## Appendix

When *V*(*x*,*t*) = 0, the right derivative of *W* is calculated by

(43)
$$\begin{array}{l} D^{+}W=\lim\;\underset{h\to 0^{+}}{\sup}\;\frac{1}{h}\left[W\left(t+h,x\left(t+h\right)\right)-W\left(t,x\left(t\right)\right)\right]\\ =\lim\;\underset{h\to 0^{+}}{\sup}\;\frac{1}{h}\sqrt{V\left(t+h,x\left(t+h\right)\right)}\;. \end{array}$$

Also we have

(44)
$$V\left(t+h,x\left(t+h\right)\right)\leq \frac{1}{2}{\left\|X\left(t+h\right)\right\|}^{2}.$$

Considering definition (19), as shown in Section 4.2, the closed‐loop error systems in the presence of uncertainties can be rearranged as

(45)
$$\dot{X}=f\left(X\right)+Bu_{d}\left(t\right),$$

where

(46)
$$f\left(X\right)=\left[\begin{array}{l} \left(a-bk\right)x-bxe\\ -\bar{c}e+\bar{b}x^{2} \end{array}\right],B=\left[\begin{array}{l} 0\\ 1 \end{array}\right].$$

When *V* (*x*,*t*) = 0, considering Equation (46) we obtain

(47)
$$X\left(t+h\right)=h\left[f\left(0\right)+Bu_{d}\left(t\right)\right]+o\left(h\right).$$

Consequently, we obtain

(48)
$${\left\|X\left(t+h\right)\right\|}^{2}=h^{2}\;{\left\|B\;u_{d}\left(t\right)\right\|}^{2}+h\;o\left(h\right).$$

Considering Equations (20) and (48), we have

(49)
$$\frac{1}{h^{2}}V\left(t+h,x\left(t+h\right)\right)\leq \frac{1}{2}{\left\|u_{d}\left(t\right)\right\|}^{2}+\frac{o\left(h\right)}{h}$$

In other words

(50)
$$\lim\;\underset{h\to 0^{+}}{\sup}\frac{1}{h}\sqrt{V\left(t+h,x\left(t+h\right)\right)}\leq \sqrt{\frac{1}{2}}\;\left\|u_{d}\left(t\right)\right\|.$$

Thus

(51)
$$D^{+}W\leq \frac{1}{\sqrt{2}}\left\|u_{d}\left(t\right)\right\|.$$

Hence for all value of *V* (*X*(*t*),*t*) we have

(52)
$$D^{+}W\leq -\gamma W+\frac{1}{\sqrt{2}}\left\|u_{d}\left(t\right)\right\|.$$
